# Effects of a gemcitabine plus platinum regimen combined with a dendritic cell-cytokine induced killer immunotherapy on recurrence and survival rate of non-small cell lung cancer patients

**DOI:** 10.3892/etm.2014.1574

**Published:** 2014-02-21

**Authors:** MIN ZHAO, HONGBING LI, LEI LI, YIJIE ZHANG

**Affiliations:** Department of Respiratory Medicine, Henan University Huaihe Hospital, Kaifeng, Henan 475000, P.R. China

**Keywords:** non-small cell lung cancer, gemcitabine plus platinum regimen, dendritic cell-cytokine induced killer cell therapy, recurrence rate, survival rate

## Abstract

The aim of the present study was to investigate the effects of a gemcitabine plus platinum (GP) regimen combined with dendritic cell-cytokine induced killer (DC-CIK) immunotherapy on the recurrence and survival rate in patients with non-small cell lung cancer (NSCLC). Patients (n=157) with stage III NSCLC that had received surgery were randomly divided into a control group and an observation group. The control group was administered with a GP regimen and the observation group received GP chemotherapy that was based on DC-CIK cell immunotherapy in addition to SC-CIK cell immunotherapy here. The two groups were followed up for 36 months and their postoperative cellular immune function, disease-free survival time, cumulative recurrence rate and cumulative survival rate was analyzed. The percentages of CD3^+^CD4^+^ T lymphocytes, natural killer cells and the CD4/CD8 ratio were identified to be significantly increased following treatment compared with those observed prior to treatment in the control and observation groups; conversely, the CD3^+^CD8^+^ T lymphocyte percentage decreased significantly (P<0.05). Furthermore, the results of the patients in the observation group were significantly better compared with the control group based on these indicators (P<0.05). The median disease-free survival time of patients in the observation group (28 months) was identified to be significantly longer than that of the control group (22 months; P<0.05), the three-year cumulative recurrence rate in the observation group (47.37%) was significantly lower than that of the control group (76.92%; P<0.05) and the three-year cumulative survival rate of the patients in the observation group (58.23%) was significantly higher than that of the control group patients (37.14%; P<0.05). In conclusion, the GP regimen combined with DC-CIK immunotherapy significantly improved the immune cell function in the postoperative NSCLC patients, in addition to reducing postoperative tumor recurrence and prolonging the survival time of patients with NSCLC.

## Introduction

Lung cancer is a common type of malignant tumor, which seriously impacts human health and quality of life due to its high incidence and mortality rates (1). Approximately 80–85% of lung cancers are non-small cell lung cancers (NSCLCs) and the majority of those patients are diagnosed at the middle or late stage, which results in a poor prognosis (2,3). Surgery is the predominant treatment method for NSCLC, however, 60–70% of patients who receive surgery exhibit postoperative recurrence and metastasis, and only 20% survive (4,5). Therefore, the aim of current clinical research is to reduce the recurrence rate and postoperative metastasis, as well as elongate the survival time of patients. Chemotherapy and radiotherapy, which have been adopted as basic postoperative treatment strategies in NSCLC, reduce the local recurrence rate in patients and extend their survival time to a certain extent, however, the effect is limited (6,7). For NSCLC patients, an effective immune system is significant for the suppression of tumor recurrence and metastasis. As a result of this, dendritic cell-cytokine induced killer (DC-CIK) cell-based immunotherapy has been widely used in numerous tumor treatments and has significantly prolonged patient survival time and improved immune function (8–10). However, the effect of DC-CIK cell immunotherapy on NSCLC, particularly stage III NSCLC, has not been reported. Post-surgery NSCLC patients received GP chemotherapy and DC-CIK cell immunotherapy in the present study.

## Patients and methods

### General information

Patients with stage III NSCLC (n=157) received complete resection surgery between June 2010 and June 2013. The inclusion criteria for the present study were as follows: i) Patients were pathologically diagnosed with adenocarcinoma, squamous cell carcinoma or adenosquamous-mixed NSCLC; ii) patients were at stage IIIA according to the International Union Against Cancer NSCLC criteria (7); iii) patients were aged 30–78 years; iv) patients exhibited normal function of the hematopoietic system, liver, kidney and heart; v) Karnofsky performance status score of the patients was >60 points; and vi) patients provided a signed informed consent sheet. The exclusion and rejection criteria were as follows: i) Those who were not able to complete the entire course of treatment and ii) patients who exhibited an adverse reaction to treatment. The 157 cases were randomly divided into a control group and an observation group. The control group consisted of 78 cases, including 46 males and 32 females (aged, 30–76 years; average age, 58.2±11.2 years). The cytology types were as follows: Adenocarcinoma (n=41), squamous cell carcinoma (n=28) and adenosquamous carcinoma (n=9). The observation group consisted of 79 cases, including 49 male and 30 females (aged, 32–78 years; average age, 59.6±10.7 years). The cytology types were as follows: Adenocarcinoma (n=44), squamous cell carcinoma (n=27) and adenosquamous carcinoma (n=8). There was no significant difference identified regarding age, gender or cytology type between the control and observation groups. The present study was conducted in accordance with the declaration of Helsinki and approval was obtained from the Ethics Committee of Henan University Huaihe Hospital (Kaifeng, China). Written informed consent was obtained from all of the participants.

### DC and CIK cell expansion

The mononuclear cells of the patients in the observation group were isolated using a blood cell separator (StatSpin, Norwood, MA, USA). Following purification using a lymphocyte separation medium, the cells were cultured for 2 h to allow pre-adhesion. The non-adherent cells were collected and the adherent cells were treated with RPMI-1640 medium containing 1,000 U/ml granulocyte-macrophage colony-stimulating factor, 500 U/ml interleukin (IL)-4 and 500 U/ml tumor necrosis factor (TNF)-α (R&D Systems, Minneapolis, MN, USA), and the medium was replaced every four days. The cells were cultured for 8–12 days and were considered to be DCs. The non-adherent cells were cultured at a concentration of 2.5×10^6^ cells/ml and treated with 1,000 U/ml interferon (INF)-γ (R&D Systems). One day later the cells were stimulated with 100 U/ml IL-1α, 500 U/ml IL-2 and 50 ng/ml cluster of differentiation (CD) 3 monoclonal antibodies (R&D Systems), and the medium was changed every three days. The cells were cultured for 8–12 days and were considered to be CIK cells. The cultured DCs and CIK cells were collected on day nine, mixed at a ratio of 1:5, co-cultured for three days and used for reinfusion. Prior to cell reinfusion, the expression level of CD80, 83, 86, 1A and human leukocyte antigen (HLA)-DR (eBioscience, SanDiego, CA, USA) in the DCs was detected using flow cytometry (BD Biosciences, Franklin Lakes, NJ, USA). In addition, the expression of CD3, 56 and 8 (eBioscience, San Diego, CA, USA) in the CIK cells was analyzed.

### Regimen

The control group patients were treated with gemcitabine plus platinum (GP) following surgery as follows: Gemcitabine (1,000 mg/m^2^), dissolved in 500 ml 5% glucose and intravenously infused at Day 1 and Day 8; 30 mg/m^2^ cisplatin (Zhunzi H20010743; Jiangsu Stockhausen Pharmaceutical Co., Ltd., Jiangsu, China) dissolved in 500 ml of 5% glucose and intravenously injected at Days 1–3. The treatment cycle was 21 days and each patient underwent four cycles of treatment. The patients in the observation group received two cycles of GP chemotherapy following surgery, at two-week intervals and subsequently received DC-CIK cell therapy twice a week. Following this, the patients received two cycles of GP chemotherapy, at two-week intervals and received a further DC-CIK cell treatment. In total, the patients in the observation group underwent four cycles of GP chemotherapy and two cycles of DC-CIK treatment.

### Observation indicators and evaluation criteria

Analysis of immune function was performed as follows: Blood (5 ml) was collected from the patients in the control and observation groups prior to and one month following treatment. The percentage of CD3^+^CD4^+^ and CD3^+^CD8^+^ T lymphocytes and CD56^+^ natural killer (NK) cells in the peripheral blood, was analyzed using flow cytometry to assess the cellular immune capacity of the patients. The two groups were followed up for 36 months to observe recurrence and metastasis. Disease-free survival time, cumulative recurrence rate and cumulative survival rate were calculated in the two groups. The time between surgery and recurrence or metastasis was considered to be the disease-free survival time (11).

### Statistical analysis

The data were analyzed using SPSS 17.0 statistical software (SPSS Inc., Chicago, IL, USA) and the measurement data were compared using Student’s t-test and were shown as means ± standard deviation. The count data were compared using a χ^2^ test and the cumulative recurrence rates, and cumulative survival rates were compared using Kaplan-Meier curves and the log-rank test. P<0.05 was considered to indicate a statistically significant difference.

## Results

### DC-CIK in vitro amplification

**Figure 1:**
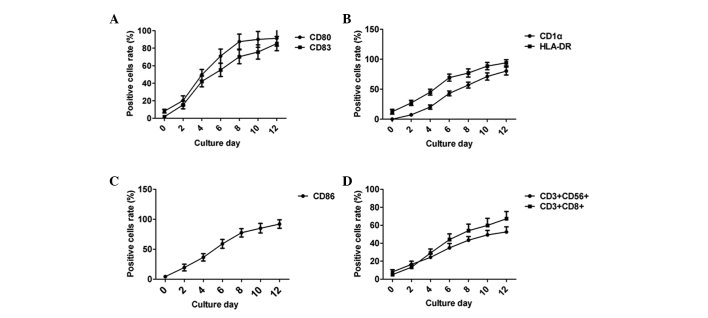
Phenotypic analysis of dendritic and cytokine induced killer cells in vitro. (A) Cell surface markers, CD80, 83. (B) Cell surface markers, CD1α and HLA-DR. (C) Cell surface marker, CD86. (D) CD3^+^CD56^+^ and CD3^+^CD8^+^ double positive cells. CD, cluster of differentiation; HLA-DR, human leukocyte antigen-DR. CD, cluster of differentiation; HLA-DR, human leukocyte antigen-DR.

The DCs were amplified *in vitro* in accordance with their culture time and the positive expression rates of the DC surface markers, CD80, 83, 86, 1α and HLA-DR, gradually increased (Fig. 1A). The number of CD3^+^CD56^+^ and CD3^+^CD8^+^ double positive CIK cells increased in accordance with their incubation time (Fig. 1B).

### Comparison of postoperative cellular immune function

**Figure 2:**
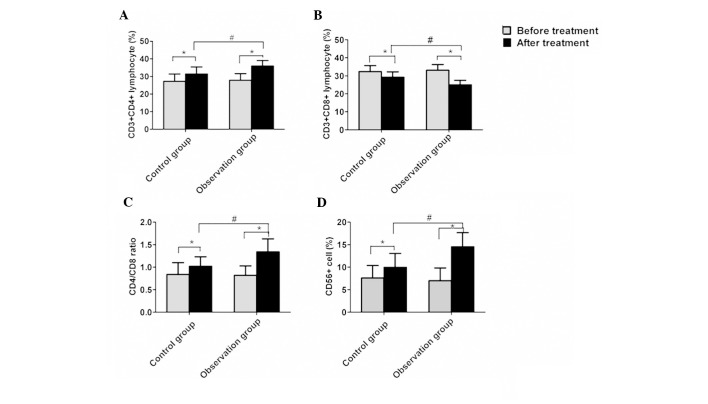
Comparison of cell immune function in the control and observation groups prior to and following treatment. (A) CD3^+^CD4^+^ T lymphocytes. (B) CD3^+^CD8^+^ T lymphocytes. (C) CD4/CD8 ratio. (D) CD56^+^ natural killer cells. ^*^P<0.05 compared with before treatment and ^#^P<0.05 compared with the control group after treatment.

The expression of CD3^+^CD4^+^ T lymphocytes, the CD56^+^ NK cell percentage and the CD4/CD8 ratio were observed to be significantly higher following treatment compared with prior to treatment in the control and observation groups, and the percentage of CD3^+^CD8^+^ T lymphocytes significantly decreased (Fig. 2, P<0.05). The increase of the CD3^+^CD4^+^ T lymphocytes, the CD56^+^ NK cell percentage and the CD4/CD8 ratio, and the reduction of the CD3^+^CD8^+^ T lymphocyte percentage in the peripheral blood were identified to be significantly increased in the observation group compared with the control group (P<0.05).

### Comparison of the cumulative recurrence rate and the cumulative survival rate

**Figure 3:**
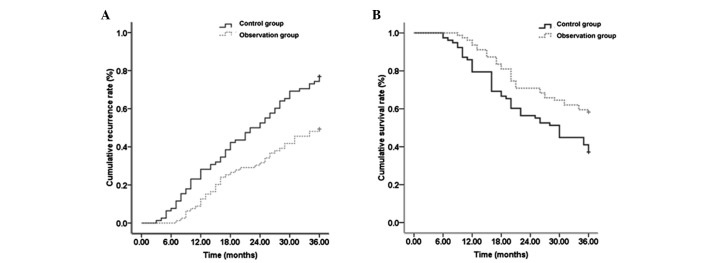
Comparison of (A) cumulative recurrence rate and (B) survival rate in the control and observation groups.

In the 36 month follow-up, the median survival time of the patients without disease in the control and observation group was 22 months (95% confidence interval (CI): 16.230–27.770 months) and 28 months (95% CI: 24.390–31.610 months), respectively, which was identified to be a statistically significant difference (P<0.05). The cumulative recurrence rates over the three years that were observed in the control group were 28.21, 52.56 and 76.92% compared with 11.39, 30.38 and 47.37% in the observation group. This identified that the cumulative recurrence rates in the observation group were significantly lower than those of the control group (Fig. 3A; P<0.05). In addition, the cumulative survival rates over the three years that were observed in the control group were 6.33, 29.11 and 58.23% compared with 37.14, 41.03 and 62.82% in the observation group. This demonstrated that the cumulative survival rates in the observation group were significantly higher than that of the control group (Fig. 3B, P<0.05).

## Discussion

Previous studies have shown that in patients with stage IIIA NSCLC, the median survival time and five-year survival rates of patients that underwent surgery were similar to that of the patients that were treated with GP chemotherapy, which indicated that surgery alone does not result in a significantly improved prognosis (12,13). Recurrence following surgery is the leading cause of mortality in patients with NSCLC, 80% of which develop distant metastases and 20% exhibit a local recurrence (14). Therefore, optimizing the postoperative treatment is critical to reduce the incidence of postoperative recurrence and, thus, extend the survival time of patients. Radiotherapy and chemotherapy are common methods of treating patients with NSCLC; however, it has been found that radiotherapy and chemotherapy do not alter the long-term outcome in the majority of stage IIIA patients, which may be associated with a plateau in the efficacy of these therapies (15). In recent years, as a result of investigation into the immune function associated with cancer development and metastasis, cellular-immunity based therapeutic strategies have been gradually applied to certain malignant types of tumors, including gastric, bladder and breast cancer, with significant effects (16–18). DCs are primary antigen-presenting cells within the body and are able to release large quantities of cytokines, such as INF and IL-12, which mediate antitumor immune responses via multiple pathways (19). CIK cells are a cell subset of heterogeneous NK cell-like cells, which originate from peripheral blood mononuclear cells and are induced by a variety of cytokines *in vivo*. The CD16^+^CD56^+^ double positive cell subset within CIK cells releases a large quantity of antitumor-associated cytokines and exhibits a strong cytotoxic effect on tumor cells independent of major histocompatibility complex II (20). Therefore, in the present study, NSCLC patients were treated with DC-CIK cell immunotherapy on the basis of a GP regimen to investigate its impact on recurrence and survival time.

The immune function of the two patient groups was analyzed in the present study. Numerous studies have shown that the immune system (particularly the cellular immune system in cancer patients) exhibits severe defects, including a reduction of CD3^+^CD4^+^ T cells, an increase in the number of CD3^+^CD8^+^ T cells, a decrease in the CD4^+^/CD8^+^ ratio and a depression of NK cell killing activity (8). The present study demonstrated that the percentage of T lymphocyte and NK cell subsets in peripheral blood were severely abnormal in the observation and control group patients. The number of CD3^+^CD4^+^ T cells, the NK cell percentage and the CD4^+^/CD8^+^ ratio were significantly higher in the control and observation groups following treatment compared with before treatment; conversely, the CD3^+^CD8^+^ T cell percentage was significantly decreased. Furthermore, the immune function of the observation group patients following treatment was significantly improved compared with the control group patients. The results indicated that immune function was restored in the DC-CIK cell-therapy treated patients. Enhancement of immune capacity considerably improved the antitumor immune response, which efficiently killed the residual tumor cells following surgery, in addition to effectively preventing tumor metastasis and recurrence, as well as prolonging the survival time of patients.

The present study further analyzed the effects of DC-CIK cell therapy combined with GP chemotherapy on NSCLC recurrence and survival time. The data demonstrated that the median disease-free survival time of patients that underwent GP chemotherapy alone was significantly shorter than that of patients who received GP chemotherapy combined with DC-CIK cell therapy. The three-year recurrence rate in the patients that received GP chemotherapy combined with DC-CIK cell therapy (47.37%) was lower than that observed in the GP chemotherapy alone group (76.92%), while the three-year cumulative survival rate of the patients that received GP chemotherapy combined with DC-CIK cell therapy (58.23%) was higher compared with the GP chemotherapy-alone group (37.14%). The results demonstrated that DC-CIK cell immunotherapy combined with GP chemotherapy was significant in reducing recurrence and extending the survival time of patients with NSCLC.

In conclusion, DC-CIK cell immunotherapy combined with GP chemotherapy improved the postoperative immune function in patients with NSCLC, reduced recurrence and extended the patient survival time following surgery; therefore, it may be adopted in the future for clinical use.

## References

[1] Siegel R, Naishadham D, Jemal A (2012). Cancer statistics, 2012. CA Cancer J Clin.

[2] Goldstraw P, Ball D, Jett JR, Le Chevalier T, Lim E, Nicholson AG, Shepherd FA (2011). Non-small-cell lung cancer. Lancet.

[3] Naim Younes R, Gross JL, Abrao FG, Rodrigues Pereira J (2013). Impact of adjuvant chemotherapy in completely resected stage IIIA non-small cell lung cancer. Minerva Chir.

[4] Filipits M, Pirker R (2011). Predictive markers in the adjuvant therapy of non-small cell lung cancer. Lung Cancer.

[5] Jemal A, Murray T, Ward E (2005). Cancer statistics, 2005. CA Cancer J Clin.

[6] Pisters KM (2005). Adjuvant chemotherapy for non-small-cell lung cancer - the smoke clears. N Engl J Med.

[7] Ost D, Goldberg J, Rolnitzky L, Rom WN (2008). Survival after surgery in stage IA and IB non-small cell lung cancer. Am J Respir Crit Care Med.

[8] Zhong GC, Yan B, Sun Y (2012). Clinical efficacy of immunotherapy of dendritic cell and cytokine-induced killer cell combined with chemotherapy for treatment of multiple myeloma. Zhonghua Xue Ye Xue Za Zhi.

[9] Zhan HL, Gao X, Pu XY, Li W, Li ZJ, Zhou XF, Qiu JG (2012). A randomized controlled trial of postoperative tumor lysate-pulsed dendritic cells and cytokine-induced killer cells immunotherapy in patients with localized and locally advanced renal cell carcinoma. Chin Med J (Engl).

[10] Shi SB, Ma TH, Li CH, Tang XY (2012). Effect of maintenance therapy with dendritic cells: cytokine-induced killer cells in patients with advanced non-small cell lung cancer. Tumori.

[11] Sargent D, Shi Q, Yothers G, Adjuvant Colon Cancer End-points (ACCENT) Group (2011). Two or three year disease-free survival (DFS) as a primary end-point in stage III adjuvant colon cancer trials with fluoropyrimidines with or without oxaliplatin or irinotecan: data from 12,676 patients from MOSAIC, X-ACT, PETACC-3, C-06, C-07 and C89803. Eur J Cancer.

[12] Fidler MJ, Kim AW, Zusag T, Bonomi P (2009). Treatment of locally advanced non-small cell lung cancer. Clin Adv Hematol Oncol.

[13] Juretic A, Sobat H, Samija M (1999). Combined modality therapy of non-small cell lung cancers. Ann Oncol.

[14] Seder CW, Allen MS, Cassivi SD (2013). Stage IIIA non-small cell lung cancer: morbidity and mortality of three distinct multimodality regimens. Ann Thorac Surg.

[15] Carney DN (2002). Lung cancer - time to move on from chemotherapy. N Engl J Med.

[16] Yuan XK, Zhao XK, Xia YC, Zhu X, Xiao P (2011). Increased circulating immunosuppressive CD14^+^HLA-DR^−/low^ cells correlate with clinical cancer stage and pathological grade in patients with bladder carcinoma. J Int Med Res.

[17] Ren J, Di L, Song G (2013). Selections of appropriate regimen of high-dose chemotherapy combined with adoptive cellular therapy with dendritic and cytokine-induced killer cells improved progression-free and overall survival in patients with metastatic breast cancer: reargument of such contentious therapeutic preferences. Clin Transl Oncol.

[18] Shi L, Zhou Q, Wu J, Ji M, Li G, Jiang J, Wu C (2012). Efficacy of adjuvant immunotherapy with cytokine-induced killer cells in patients with locally advanced gastric cancer. Cancer Immunol Immunother.

[19] Sangiolo D, Mesiano G, Carnevale-Schianca F, Piacibello W, Aglietta M, Cignetti A (2009). Cytokine induced killer cells as adoptive immunotherapy strategy to augment graft versus tumor after hematopoietic cell transplantation. Expert Opin Biol Ther.

[20] Zheng YW, Li RM, Zhang XW, Ren XB (2013). Current adoptive immunotherapy in non-small cell lung cancer and potential influence of therapy outcome. Cancer Invest.

